# Impact of Demographics and Psychological Factors on Three-Day Postoperative Pain Perception Following Hand Surgery

**DOI:** 10.3390/jcm14010037

**Published:** 2024-12-25

**Authors:** Sahar Borna, Olivia A. Ho, Cesar A. Gomez-Cabello, Syed Ali Haider, Ariana Genovese, Srinivasagam Prabha, Clifton R. Haider, Christopher L. Felton, Christopher J. McLeod, Charles J. Bruce, Rickey E. Carter, Antonio Jorge Forte

**Affiliations:** 1 Division of Plastic Surgery, Mayo Clinic, Jacksonville, FL 32224, USA; 2 Department of Physiology and Biomedical Engineering, Mayo Clinic, Rochester, MN 55905, USA; 3 Department of Cardiovascular Medicine, Mayo Clinic, Jacksonville, FL 32224, USA; 4 Department of Biomedical Statistics and Informatics, Mayo Clinic, Jacksonville, FL 32224, USA; 5 Center for Digital Health, Mayo Clinic, Rochester, MN 55905, USA

**Keywords:** postoperative pain, anxiety, catastrophization, pain measurement, acute pain, sex, age

## Abstract

**Background:** Effective pain management is crucial for both comfort and outcomes, yet predicting and managing this pain is difficult. This study aimed to analyze postoperative pain in patients undergoing hand surgery at the Mayo Clinic Florida, examining how patient characteristics and anxiety affect pain outcomes. **Methods:** We conducted a single-arm clinical trial at Mayo Clinic Florida, recruiting patients undergoing hand surgery. Preoperative pain and anxiety were assessed using the Pain Catastrophizing Scale (PCS) and State-Trait Anxiety Inventory (STAI). Postoperatively, patients used an iPhone app to record pain levels and medication use every four hours. Devices were collected three days after surgery. We analyzed the relationship between demographics, PCS, STAI scores, and pain levels using linear and logistic regression models. All statistical tests were two-sided with significance set at *p* < 0.05, analyzed with R4.2.2. **Results:** Data were collected from 62 patients (62.9% women, 37.1% men) undergoing hand surgery. Participants were mainly White (90.3%), with 50% being in the middle-aged adult group. Most had low anxiety levels (80.6% STAI-S, 82.3% STAI-T) and low catastrophizing (61.3% PCS). Postoperative pain was low, with median scores between 1.0 and 2.0 over three days. Demographics, anxiety, and catastrophizing were not significant predictors of pain levels. Logistic regression showed time as a significant factor, with pain levels peaking on Day 3. **Conclusions:** Postoperative pain after hand surgery was generally low, with time being a significant predictor of increased pain. Demographic factors, anxiety, and catastrophizing did not significantly affect pain levels. Pain management should emphasize time-sensitive interventions and ongoing monitoring.

## 1. Introduction

Each year, nearly 100 million surgeries are performed in the United States according to statistics from the Centers for Disease Control and Prevention (CDC) [1,2]. A nationwide survey revealed that over 80% of patients experience acute postoperative pain, with 75–86% of these individuals reporting pain levels that exceed moderate intensity [1,3]. Postsurgical pain (PSP) is a prevalent and significant concern, with nearly 60% of patients expressing anxiety about pain management following their procedures [3].

Effective pain management is essential for patient comfort, satisfaction, and better surgical outcomes [4]. Pain management strategies span a spectrum of interventions, from accurate pain prediction to advanced techniques such as patient-controlled analgesia (PCA), transdermal therapeutic systems (TTSs), and regional anesthesia techniques (epidural/subarachnoid block) [4,5]. In orthopedic surgery, more than 98% of American patients are prescribed opioid medications, underscoring the need for more efficient pain management approaches that could reduce opioid use [6].

Preoperative predictors of postoperative pain (PSP) encompass a range of factors, including patient characteristics such as age, sex, and body mass index (BMI), as well as psychological factors, pre-existing pain, the type of surgery, and wound size [7]. Two commonly used tools for assessing preoperative psychological status are the Pain Catastrophizing Scale (PCS) and the State-Trait Anxiety Inventory (STAI). These tools measure different aspects of psychological functioning that can influence pain perception. The State-Trait Anxiety Inventory, developed by Spielberger, evaluates anxiety through two distinct scales: A-State and A-Trait. The A-State scale (STAI-S) assesses current feelings of tension and nervousness, while the A-Trait scale (STAI-T) measures general anxiety tendencies. Each scale consists of 20 self-reported statements, providing a comprehensive evaluation of the patient’s anxiety profile [8]. Research has demonstrated that both the anxiety and catastrophizing levels measured by these scales are positively correlated with pain intensity, including in emergency settings [9,10]. The PCS is a well-established self-report instrument designed to measure pain-related catastrophizing. This scale evaluates how individuals perceive and react to pain, focusing on their tendency to ruminate, magnify, or feel helpless about pain. Evidence suggests that the PCS is a strong predictor of pain intensity, independent of anxiety levels [9,11,12]. Its reliability and specificity make it a valuable tool for identifying patients who may experience heightened postoperative pain.

Pain perception is influenced by various factors, including the type of stimulus (such as heat versus pressure) and the specific neuroreceptors involved [13]. Clinical research shows that race contributes to variations in pain sensitivity, and aging leads to significant neurochemical and neuroanatomical changes that affect pain sensitivity, notably reducing responses to thermal and electric shock stimuli and correlating with fewer opioid receptors in the brain [14,15]. Notably, this diminished sensitivity tends to be more pronounced in women than in men. In clinical settings, older patients have been observed to perceive less severe and more delayed pain from myocardial ischemia compared to younger individuals [16]. However, the effect of sex on pain perception remains contentious, with research producing mixed results. This highlights the necessity of considering a range of factors including cultural, physiological, and psychological elements when evaluating pain responses [17].

In this study, we analyzed postoperative pain levels among patients who underwent hand surgery at the Mayo Clinic Florida’s departments of Plastic Surgery and Orthopedic Surgery. Our objective was to explore the relationship between patient characteristics and pain levels, with the aim of improving pain management strategies.

## 2. Materials and Methods

### 2.1. Patient Recruitment and Data Collection

In this single-arm prospective clinical trial conducted at Mayo Clinic Florida, we enrolled patients aged 18 and older who underwent minor hand surgeries between November 2022 and March 2024. Recruitment took place in the departments of Plastic Surgery and Orthopedic Surgery. The study included 51 distinct hand surgery procedures, such as carpal tunnel release, trigger finger release, and Dupuytren’s fasciectomy under general anesthesia, wide awake local anesthesia, and peripheral nerve blocks, depending on the surgeon’s discretion and the specific requirements of each procedure. Our sample size calculation was performed by using the online calculator CliniCalc. The data we used are based on a study by Pouromran et al. and we considered the mean absolute error for their best-performing autonomic variable (0.93 ± 0.19) as the known mean in our equation [18]. In our study, to detect a root mean square error of at least 1 unit with a two-tailed alpha of 0.05 and a power of 80%, we needed data from at least 58 patients. We therefore recruited 100 patients. Exclusion criteria included patients with severe allergies to medical glue, communication barriers, inability to take oral medication, use of nerve catheters for pain control, chronic pain treatment for over six months, severe anxiety disorders like PTSD, baseline immobility, and pregnancy.

All patients received standard perioperative analgesia. The Institutional Review Board of Mayo Clinic approved the study protocol and participant consent forms (IRB protocol number 21-013443). For postoperative pain management, patients who did not receive a blocking catheter were provided with an OMNI device developed by Mayo Clinic to monitor physiological variables and an iPhone with the OMNI application. The app enabled patients to record their pain levels using a numerical rating scale (NRS) from 1 to 10 every four hours during their awake hours (8 a.m. to 8 p.m.) and to log their medication intake, including type and amount. The OMNI device and iPhone were collected from patients three days after surgery via prepaid FedEx, with all data anonymized.

The primary objective of this analysis was to assess the relationship between patient demographics, PCS and STAI scores, and reported pain levels during the first four days after surgery.

### 2.2. Statistical Analysis Methods

Continuous variables are summarized as median and mean. Categorical data are reported as frequency. STAI scores were grouped as follows: 1 for low anxiety (scores below 40), 2 for moderate anxiety (scores between 40 and 54), and 3 for high anxiety (scores above 54). PCS scores were divided into four groups: 1 for Low catastrophization (scores 0 to 9), 2 for moderate catastrophization (scores 10 to 19), 3 for high catastrophization (scores 20 to 39), and 4 for very high catastrophization (scores 40 to 52). Fixed-effects linear models were used to predict pain levels on each postoperative day, with demographic, PCS, and STAI scores as predictors. Mixed-effects linear models were also used to explore the association between demographic data, PCS and STAI scores, and pain levels over 4 days after surgery (including the day of surgery). In this model, demographic factors, PCS, STAI scores, and postoperative days were used as fixed effects, and a random slope was allowed for each patient. As an alternative approach, pain scores were also dichotomized as ≤3 and >3, and mixed-effects logistic regression models were used to evaluate the same fixed effects as mentioned above. All tests were two-sided, with a *p*-value < 0.05 considered statistically significant. The analysis was conducted using R4.2.2.

## 3. Results

We successfully recruited 100 patients, of whom 62 completed the forms correctly and were eligible to participate in this study. STAI scores indicated that most participants had low levels of anxiety, with 80.6% (n = 50) scoring in the lowest category (score 1) for STAI-S and 82.3% (n = 51) scoring in the lowest category for STAI-T. For STAI-S, 17.7% (n = 11) scored in the second category and 1.6% (n = 1) in the third category. For STAI-T, 17.7% (n = 11) scored in the second category.

The PCS results showed that 61.3% (n = 38) of the participants had low levels of catastrophizing (score 1), while 30.6% (n = 19) and 8.1% (n = 5) fell into higher categories (scores 2 and 3, respectively). Detailed results are presented in Table 1, Table 2, Table 3, Table 4 and Table 5.

The mixed-effects model allowed us to examine the pain scores over 4 days, with time as another predictor. The only significant predictor for pain was time, with the pain score being significantly higher on day 1 compared to day 0.

Due to the skewed distribution of the pain scores, we employed a logistic regression model to better examine the relationship between pain levels and other variables. In this model, we categorized the pain levels into two groups: scores of 1–3 were considered low pain, while scores greater than 3 were classified as high pain.

## 4. Discussion

### 4.1. Effects of Demographics on Pain Perception

The research on sex differences in pain perception presents mixed findings. Some studies indicate that women report pain differently from men, yet the overall intensity of self-assessed pain does not vary significantly among the sexes. Conversely, other studies show that women experience higher pain intensity and unpleasantness than men, with notable differences in pain location; for example, women report more pain on the face compared to the ankle under similar stimuli [19,20]. Additionally, women tend to have higher sensitivity to experimentally induced pain and report greater clinical pain and distress [21]. Our study found no significant sex-based differences in pain levels, whether in routine or severe pain assessments (levels above three), either on the day of surgery or up to three days after surgery. This aligns with a systematic review that did not identify a consistent pattern of pain perception differences among sexes. However, it is worth noting that the type of pain—such as pressure, thermal, chemical, or electrical—can influence perceptions, with pressure pain being reported higher in women [22]. Similarly, another study found no sex-based differences in pain perception based on age, sex, or ethnicity but used the Hospital Anxiety and Depression Scale (HADS) and focused only on the first 24 h after surgery, potentially skewing results due to analgesic use [23]. While some studies report higher pain levels in women, especially after cardiothoracic and neurosurgical procedures, this discrepancy was not significant in cases of oral surgery [24]. Studies have identified various factors contributing to the inconsistent results in pain perception, including genetics. However, these factors alone cannot fully explain the sex differences in pain perception. Other significant factors include variations in central pain processing systems and gonadal hormones. Additionally, responses to opioid medications differ among the sexes, with women often experiencing greater analgesic effects from opioids and thus requiring lower doses compared to men. In contrast, nonopioid analgesic effects do not differ significantly among the sexes [21]. Reviews indicate that larger studies often reveal more pronounced differences in pain perception between sex compared to smaller, less consistent studies [24].

We categorized participants into three age groups: young adults (18–40 years), middle-aged Adults (41–60 years), and senior adults (61 years and above). In contrast to other studies, our results show no significant difference in pain levels across these age groups. The existing research indicates that postoperative pain tends to decrease by 0.28 NRS points per decade of age, while conditioned pain modulation (CPM) declines with age [24]. A meta-analysis of 31 studies on age-related pain perception supports these conclusions, showing that older individuals often exhibit decreased pain sensitivity at lower pain levels, suggesting higher pain thresholds as age increases. This analysis carefully considered the publication dates since the majority of the studies are older, potentially rendering their findings less reliable due to improved living conditions for older adults and changes in pain induction mechanisms [25]. However, studies showed the impact of aging is more pronounced under heat stress compared to pressure or electrical stimuli [25]. This suggests that the type of pain plays a critical role in age-related pain studies. While our experiment focused on noxious stimuli, which may not directly correlate with findings from studies on non-noxious stimuli, pressure pain thresholds, which are applicable to our study, decrease with age [13,26]. Changes in pain perception can vary across different layers of pain processing; for instance, skin nociception may differ from muscular nociception with advancing age [13]. The research indicates that a reduction in peripheral nerves and supporting tissues with age contributes to decreased sensory sensitivity [14]. This variation in pain perception with age can be attributed to a range of factors, including anatomical-, physiological-, age-related plasticity; immune system changes; neuroendocrine alterations; inflammation; and autonomic responses [14]. However, the limited number of studies specifically addressing age-related changes in pain tolerance thresholds constrains our ability to draw definitive conclusions [25].

Additionally, pain levels can vary depending on the type of surgery, and numerous confounding variables must be considered. In our study, we adjusted for factors such as patient anxiety, race, and sex to improve result accuracy despite the limited sample size. Even within orthopedic procedures, pain perception can differ based on the surgical site, with certain surgeries, such as total hip arthroplasty, showing higher pain levels in women [24]. Recognizing and addressing these demographic disparities is crucial for tailoring patient management and improving satisfaction. Such insights can guide policymakers and healthcare providers in customizing care to meet diverse patient needs more effectively.

### 4.2. Impact of Preoperative Anxiety on Postoperative Pain

Catastrophizing has been shown to be a critical predictor of postoperative pain across various clinical settings. By using both the PCS and STAI scales, our study aimed to understand the role these psychological factors play in influencing postoperative pain [27,28]. Research has long established catastrophizing as a critical predictor of postoperative pain in various contexts, ranging from chronic pain in clinical settings to acute pain tolerance in non-clinical scenarios [21]. The use of both scales in our study allowed us to explore how these psychological factors influence pain perception in a diverse surgical population.

Preoperative anxiety has been consistently linked to increased postoperative pain in various surgical contexts. For instance, Daştan et al. found that hand massage to reduce anxiety before cataract surgery led to significantly lower postoperative pain [29]. Similarly, Zhang et al. demonstrated that occupation and previous surgical experience can influence preoperative anxiety levels in women undergoing laparoscopic hysterectomies. They reported that higher preoperative anxiety was associated with increased postoperative pain [30], a finding echoed in studies of elective abdominal surgeries and confirmed by Tadesse et al., who observed this relationship up to 12 h after surgery [9,31]. Bradshaw et al. observed a 43% prevalence of anxiety in their patient population, which correlated with increased postoperative pain. Similar to our study, they differentiated patients based on broader surgical specialties, such as general surgery or orthopedic surgery, instead of focusing on specific types of surgical procedures. This observation aligns with the approach in the Granot et al. study, where they exclusively included patients undergoing abdominal surgery, without distinguishing between specific types of procedures within that category [9,23]. In contrast to studies like that of Suresh et al., which focused solely on women undergoing elective cesarean sections, our study included patients of different sexes, races, and surgical types. This diversity offers a broader perspective on how demographic factors and procedural variability influence the relationship between anxiety and postoperative pain, enhancing the generalizability of our results [32]. Mimic et al. also found a strong correlation between preoperative anxiety and postoperative pain in patients undergoing open nephrectomy [33]. Using additional tools like the Amsterdam Preoperative Anxiety and Information Scale (APAIS) and the Hamilton Anxiety Rating Scale (HAM-A), they offered a multidimensional view of anxiety’s impact. Interestingly, they found that expected pain predicted immediate postoperative pain, while anxiety and catastrophizing became more significant in the late postoperative phase. Our study, which also used the PCS, similarly found no significant predictive role for demographic factors like age, sex, or race, aligning with Mimic et al.’s findings [33]. Similarly, a study by Suresh et al. involved patients undergoing laparoscopic cholecystectomy, and they used the STAI-T and STAI-S to assess anxiety and evaluate pain using a visual analog scale (VAS). Despite the methodological differences, both studies were conducted at single centers [34]. Across different surgical procedures, the relationship between preoperative anxiety and postoperative pain has been inconsistent. Some studies, such as those involving ear, nose, and throat surgeries [35] and cesarean sections [36], found a significant correlation, which suggests that for some types of surgeries, anxiety plays a clear role in how patients perceive pain postoperatively. However, other studies, like one examining patients undergoing pectus excavatum correction, found no significant association [37]. One key methodological difference that could account for these discrepancies is how anxiety was assessed. For example, Kavakcı et al. did not distinguish between state and trait anxiety, which may have limited their ability to capture the complexities of anxiety’s influence on pain perception [35]. Additionally, the Kavakcı et al. study involved patients undergoing different types of surgeries with varying diagnoses, further complicating the interpretation of their results and limiting generalizability. As highlighted in studies examining multisurgical cohorts, the inclusion of multiple surgical types allows for valuable comparisons of the postoperative pain course despite the inherent variability within each procedure [38]. For example, revision surgeries vary greatly in blood loss and complexity, yet broad patterns can still be observed in pain response due to shared injury mechanisms. Similarly, in our study, the consistent pain trajectories across different hand surgery procedures suggest a generalized postsurgical response rather than specific procedural effects [39]. While the results from patients undergoing reconstructive surgeries for lower extremity fractures [40] may align with our findings due to similarities in injury mechanisms and surgical approaches, the scarcity of studies specifically focusing on hand surgery complicates direct comparisons. This not only highlights the need for more research in this area but also underscores the significance of our study as one of the pioneering projects in this field.

**Figure 1:**
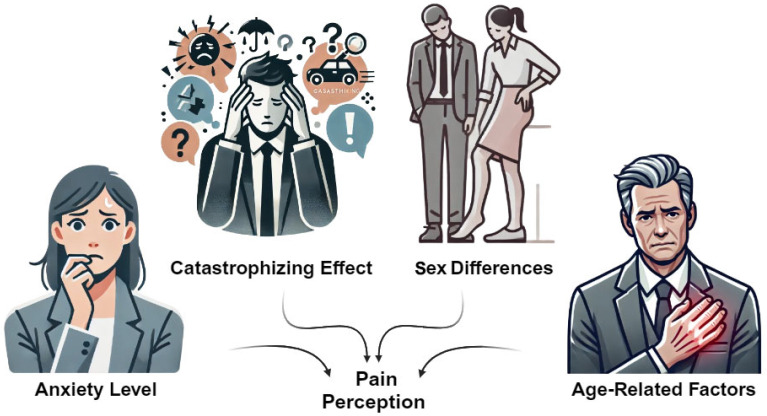
The impact of various characteristics on patients’ pain perception. Factors such as catastrophization level, preoperative anxiety, age, and sex have been shown to influence how patients perceive pain.

The discrepancies between these studies and our findings may also stem from differences in patient perceptions of their surgeries. Research suggests that patients who receive more detailed information about their procedures tend to experience less anxiety. Part of this information can be provided during the informed consent process [32,41]. This education can be delivered through various methods, such as presentations, multimodal support, and verbal instruction [42,43]. Additionally, older age often correlates with more experience in surgical and healthcare settings, which may reduce anxiety levels. For instance, Bradshaw et al. reported similar results to ours, finding that younger patients exhibited higher preoperative anxiety than older patients. However, like our study, they did not find a significant difference in postoperative pain levels between these age groups [23]. In our study, we found no significant association between preoperative anxiety, pain catastrophizing, and postoperative pain when considering factors like gender, age, ethnicity, or race. The only consistent predictor of pain was time: pain levels were significantly higher on the first postoperative day compared to the day of surgery, likely due to the waning effects of pain medication. This pattern is consistent with the natural course of postoperative recovery, where initial pain relief from anesthesia or medication wears off, leading to higher pain scores on the following day. These results can emphasize the need for enhancing monitoring by increasing the frequency of pain assessments on the first and third postoperative days and developing an early intervention protocol for when pain scores exceed a set threshold, involving adjustments in analgesics or multimodal strategies. Additionally, educating patients about the typical pain trajectory following surgery and adjusting the timing of analgesic administration can optimize pain control during peak times, as shown in Figure 1 [44].

### 4.3. Limitations and Strengths of This Review

Our study exhibits limitations that warrant consideration for future research. Primarily, the demographic composition of our sample, predominantly middle-aged and White, may not accurately reflect the diverse patient population typically encountered in hand surgery. To enhance the applicability of our findings, subsequent studies should aim to include a broader demographic spectrum, potentially employing stratified sampling techniques to evaluate whether our results hold across diverse groups. A notable strength of our study is the systematic approach to distinguishing between state and trait anxiety using the STAI. This differentiation is crucial, as state anxiety, temporary anxiety triggered by specific situations, may have a more immediate impact on pain perception compared to trait anxiety, which is indicative of an individual’s general tendency toward experiencing anxiety.

However, our study’s relatively small sample size poses a significant limitation. The modest number of participants may restrict the generalizability of our findings and diminish the statistical power needed to detect smaller yet clinically relevant effects. This issue is particularly pertinent in our analysis of the interactions between demographic and psychological factors, where high variability is anticipated. The wide confidence intervals associated with our estimates suggest a level of uncertainty that should be carefully considered when generalizing these findings to a broader population. Comparatively, the study by Suresh et al., which included a similar sample size of 66 patients, assessed postoperative pain only up to 24 h after surgery. In contrast, our study extended this follow-up period to three days, providing a more prolonged observation of pain dynamics. However, these comparisons highlight the inherent challenges in drawing comprehensive conclusions from limited datasets and underscore the necessity for larger, more diverse studies to elucidate the complex relationships between anxiety and postoperative pain more accurately. Additionally, our study did not explore the underlying causes of patients’ anxiety nor did it examine if enhanced patient education before surgery could mitigate these anxieties. Future research addressing these gaps could offer valuable insights into how effectively managing preoperative anxiety might reduce postoperative pain, thereby improving patient outcomes.

## 5. Conclusions

In this study, we explored the relationship between patient demographic factors, preoperative anxiety, and catastrophizing with perceived pain levels following hand surgery. No significant correlations were found between pain levels and patient demographics or between pain and scores from the PCS and STAI assessments. Future research should aim to include a more extensive and diverse patient population with a broader range of surgeries to enhance generalizability, particularly for more complex procedures. Additionally, further investigation into the sources of preoperative anxiety could help mitigate patient stress and improve overall management. Improving our understanding of these relationships will help optimize postsurgical care and potentially reduce the dependency on opioids.

## Figures and Tables

**Table 1:** Participant demographics (n = 62).

Category	n (%)
Sex	
Male	23 (37.1%)
Female	39 (62.9%)
Age Group	
Young Adults (18–40 years)	6 (10.0%)
Middle-Aged Adults (41–60 years)	31 (50.0%)
Senior Adults (61+ years)	25 (40.0%)
Ethnicity	
Not Hispanic or Latino	56 (90.3%)
Hispanic or Latino–South American	2 (3.2%)
Hispanic or Latino	3 (4.8%)
Not Disclosed	1 (1.6%)
Race	
White	56 (90.3%)
Black or African American	3 (4.8%)
Asian	2 (3.2%)
Not Disclosed	1 (1.6%)

**Table 2:** Summary of pain scores on each postoperative day.

Postop Day	Median (Range)	N-Miss	Mode (%)	Maximum Pain Level Observed (%)
Day 0	1.0 (1.0, 7.0)	0	1 (56.5%)	7 (3.2%)
Day 1	2.0 (1.0, 8.0)	1	2 (34.4%)	8 (1.6%)
Day 2	2.0 (1.0, 8.0)	2	1 (43.3%)	8 (1.7%)
Day 3	1.0 (1.0, 8.0)	9	1 (60.4%)	8 (1.9%)

N-Miss = number of missing observations.

**Table 3:** Univariable linear model predicting pain level on each postoperative day.

Term	Beta (95CI)	*p*-Value
Univariable models predicting pain on day 0		
Age (Reference: Young Adult 18–40)	0.01 (−0.62, 0.64)	0.97
Sex (Reference: Male)	0.2 (−0.77, 1.18)	0.69
STAI-S (High vs. Low Anxiety)	−0.11 (−1.31, 1.08)	0.85
STAI-T (High vs. Low Anxiety)	0.13 (−1.11, 1.36)	0.84
PCS (High vs. Low PCS)	0.73 (−0.22, 1.69)	0.14
Univariable models predicting pain on day 1		
Age (Reference: Young Adult 18–40)	−0.13 (−0.68, 0.42)	0.64
Sex (Reference: Male)	0.53 (−0.3, 1.37)	0.22
STAI-S (High vs. Low Anxiety)	0.49 (−0.53, 1.52)	0.35
STAI-T (High vs. Low Anxiety)	−0.15 (−1.22, 0.91)	0.78
PCS (High vs. Low PCS)	−0.24 (−1.08, 0.6)	0.58
Univariable models predicting pain on day 2		
Age (Reference: Young Adult 18–40)	−0.06 (−0.61, 0.49)	0.83
Sex (Reference: Male)	0.19 (−0.65, 1.04)	0.65
STAI-S (High vs. Low Anxiety)	0.36 (−0.7, 1.42)	0.51
STAI-T (High vs. Low Anxiety)	0.14 (−0.92, 1.2)	0.80
PCS (High vs. Low PCS)	0.23 (−0.61, 1.07)	0.60
Univariable models predicting pain on day 3		
Age (Reference: Young Adult 18–40)	−0.02 (−0.55, 0.51)	0.95
Sex (Reference: Male)	0.31 (−0.5, 1.12)	0.46
STAI-S (High vs. Low Anxiety)	0.33 (−0.66, 1.33)	0.52
STAI-T (High vs. Low Anxiety)	0.35 (−0.74, 1.43)	0.53
PCS (High vs. Low PCS)	0.67 (−0.12, 1.47)	0.10

All predictors included were prespecified. For categorical factors, the estimated mean difference in the pain score was calculated with the lowest level used as the reference. We considered the categorical new STAI-S score, and the smaller groups of scores of 2 and 3 were combined together. The same applies for the PCS score.

**Table 4:** Multivariable mixed-effects linear model predicting pain scores over 3 postoperative days.

Term	Estimated (95CI)	*p*-Value
Age (Reference: Young Adult 18–40)	−0.02 (−0.44, 0.39)	0.908
Gender (Reference: Male)	0.26 (−0.39, 0.92)	0.433
STAI-S (High vs. Low Anxiety)	0.21 (−0.82, 1.24)	0.687
STAI-T (High vs. Low Anxiety)	−0.12 (−1.23, 0.98)	0.825
PCS (High vs. Low PCS)	0.21 (−0.45, 0.87)	0.540
Time: Day 1	0.62 (0.13, 1.11)	0.015
Time: Day 2	0.02 (−0.47, 0.52)	0.924
Time: Day 3	−0.36 (−0.87, 0.16)	0.175

All predictors included were prespecified. For the categorical factors, the estimated mean difference in the pain score was calculated, with the lowest level used as the reference.

**Table 5:** Multivariable mixed-effects logistic model predicting pain scores > 3 over 3 postoperative days.

Term	OR (95CI)	*p*-Value
Model #1: Demographics, STAI, and Time		
Age (Reference: Young Adult 18–40)	1 (0.47, 2.13)	0.99
Sex (Reference: Male)	0.84 (0.27, 2.64)	0.76
STAI-S (High vs. Low Anxiety)	2.65 (0.44, 15.94)	0.29
STAI-T (High vs. Low Anxiety)	0.43 (0.06, 3.14)	0.40
Time: Day 1	2.1, (0.8, 5.5)	0.13
Time: Day 2	0.84, (0.3, 2.34)	0.74
Time: Day 3	0.26, (0.07, 0.97)	0.05
Model #2: Demographics, PCS, and Time		
Age (Reference: Young Adult 18–40)	1.14, (0.55, 2.38)	0.72
Sex (Reference: Male)	0.89, (0.29, 2.75)	0.84
PCS (High vs. Low PCS)	1.55, (0.5, 4.81)	0.45
Time: Day 1	2.09, (0.8, 5.46)	0.13
Time: Day 2	0.83, (0.3, 2.32)	0.73
Time: Day 3	0.26, (0.07, 0.98)	0.05

All predictors included were prespecified. For the categorical factors, the estimated OR was calculated with the lowest level used as the reference.

## Data Availability

The original contributions presented in this study are included in the article. Further inquiries can be directed to the corresponding author(s).
